# Mastocytose cutanée: aspect de bébé léopard

**DOI:** 10.11604/pamj.2013.16.146.3668

**Published:** 2013-12-20

**Authors:** Najwa Guerouaz, Badredine Hassam

**Affiliations:** 1 Hôpital Universitaire Ibn Sina, Service de dermatologie et vénérologie, Maroc

**Keywords:** Bébé léopard, mastocytose cutanée, signe de Darier, baby leopard, cutaneous mastocytosis, Darier's sign

## Image en medicine

Les mastocytoses correspondent à une infiltration tissulaire bénigne de mastocytes dans la peau et éventuellement dans d'autres organes. Les manifestations cliniques des mastocytoses sont variées, et liées en partie aux médiateurs libérés par les mastocytes sur un mode paroxystique. Nous rapportons le cas d'un nourrisson de 9 mois, issu d'un mariage non consanguin, sans antécédents pathologiques particuliers, qui présente depuis l’âge de 2 mois des macules brunâtres siégeant d'abord sur l'abdomen, puis se généralisant à tout le corps et évoluant par poussées. L'examen dermatologique a révélé de multiples macules grossièrement arrondies brunâtres siégeant sur tout le corps sans épargner le visage. Le signe de Darier était positif. La biopsie cutanée a montré un infiltrat mastocytocytaire dermique. Le reste du bilan était normal. La mastocytose cutanée diffuse est très rare qui survient précocement en période néonatale. Certaines particularités sont propres à l’âge de survenue. Ainsi chez l'enfant, les lésions sont volontiers de grande taille, ovalaires, de teinte brun clair, légèrement saillantes, de consistance élastique donnant parfois un aspect tigré ou “peau de léopard” comme chez notre patient. L’évolution spontanée se faisant vers la régression voire la guérison pendant l'enfance.

**Figure 1:**
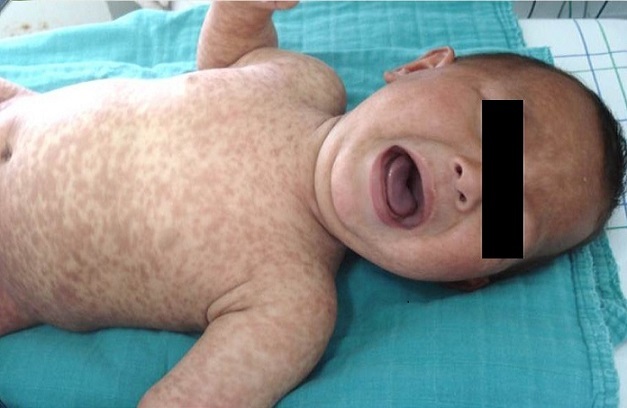
Multiples macules grossièrement arrondies brunâtres siégeant sur tout le corps

